# The pace and shape of ant ageing

**DOI:** 10.1111/brv.70035

**Published:** 2025-05-15

**Authors:** Luisa M. Jaimes‐Nino, Jan Oettler

**Affiliations:** ^1^ Institute of Organismic and Molecular Evolution Johannes Gutenberg University Hanns‐Dieter Hüsch Weg 15 Mainz 55128 Germany; ^2^ Zoologie/Evolutionsbiologie Universität Regensburg Universitätsstr. 31 Regensburg 93053 Germany

**Keywords:** lifespan, reproduction, senescence, continuusparity, social insects

## Abstract

Ants have been proposed as good models to study ageing and the effects of extrinsic mortality because of their long lifespans and plasticity of ageing within species. We discuss how age‐dependent extrinsic mortality might influence queen lifespan, and how the effect of age‐independent extrinsic mortality needs further study, accounting for different density‐dependence scenarios. Based on a critical review of the available demographic data, we discuss the selective forces underlying ant ageing. We discuss differences and similarities between the life‐history strategy of ants and the reproductive strategies iteroparity and semelparity. We consider how late‐life fitness gains for the “superorganism” select for a delay of actuarial, and reproductive senescence, and we suggest future research directions.

## I. INTRODUCTION

As part of a debate in the late 1990s over the validity of evolutionary theories explaining lifespan variation (Le Bourg, 2001), a seminal paper compared the lifespans of ants with those of solitary insect species (Keller & Genoud, 1997). Their results have become common knowledge: ant queens live longer than solitary insects, and monogynous queens live longer than polygynous queens. These differences were discussed with respect to variation in extrinsic mortality: with low mortality of the sheltered queens argued to select for long lifespans, whereas high mortality selects for earlier senescence and shorter lifespans of polygynous queens. The paper inspired a new field – social insect ageing research – which has received increasing interest ever since. So where do we stand now, 30 years later, and can ants help us understand the following three fundamental problems tackled by ageing research: why and how do organisms age; why and how do species vary in the pace of ageing (lifespan); and why does ageing vary within taxa (also known as the heterogeneity of ageing)?

## II. WHY AND HOW DO SPECIES AGE?

“Senescence”, in physiological terms, is based on structural decay or functional decline (Lemoine, 2020) resulting in a decrease in age‐specific components of fitness with increasing chronological age (Abrams, 1993). In demographic terms, “senescence” entails an increase in mortality rate (i.e. “demographic ageing”, or “actuarial senescence”), and a decrease in fertility rate (i.e. “reproductive senescence”) with age. Thus, we differentiate it from “ageing”, treated herein as all ageing‐specific changes in mortality and fertility with chronological age. To characterize age‐specific changes, two dimensions are recognized: the pace of ageing, related to the timescale on which mortality progresses (commonly measured as maximum lifespan, or life expectancy), and the shape of ageing, describing how abruptly changes in mortality and fertility rates occur (Baudisch, 2011). Senescence was first explained as resulting from a decrease in the force of natural selection with time (Medawar, 1952). Individuals producing the same amount of progeny from maturity until death increase their total progeny linearly with time. However, due to the removal of individuals in a population by extrinsic mortality (e.g. predation, disease, starvation), the contribution of offspring to the next generation decreases per age group, and therefore so does the force of natural selection; a phenomenon known as the selection shadow (Haldane, 1941; Hamilton, 1966). This can lead to late‐expressed deleterious genes and mutations being less subject to negative selection (i.e. the “mutation accumulation theory”; Medawar, 1952). Additionally, the “antagonistic pleiotropy theory” states that late‐expressed deleterious genes could fixate in a population if they are beneficial early in life (Williams, 1957; Gaillard & Lemaître, 2017). Antagonistic pleiotropy might occur *via* energy and/or functional trade‐offs. In the former case there may be limitation of resources for allocation between reproduction and somatic maintenance [i.e. “disposable soma theory” (Kirkwood, 1977; Kirkwood & Austad, 2000)], while in the latter case suboptimal gene regulation is present after maturation [i.e. the “developmental theory of ageing” (Cutler, 1979; De Magalhães & Church, 2005; Blagosklonny, 2006)].

### (1) The role of extrinsic mortality

A verbal prediction stated that the rate of senescence should decrease and average lifespan should increase as the rate of age‐ and condition‐independent extrinsic mortality decreases (Williams, 1957). However, age‐independent extrinsic mortality is insufficient to explain senescence patterns without taking into account density dependence (Hamilton, 1966; Wensink, Caswell & Baudisch, 2017; Moorad, Promislow & Silvertown, 2019; Day & Abrams, 2020). In populations where growth is density dependent, the effect of extrinsic mortality on the age‐specific selection gradient may be either positive or negative (Abrams, 1993; Caswell, 2007; Moorad *et al*., 2019; de Vries, Galipaud & Kokko, 2023). Age‐independent extrinsic mortality can affect senescence patterns if density dependence acts on fertility by favouring fast life histories (short lifespans) (Day & Abrams, 2020). For example, fast life histories are favoured if density dependence negatively affects the production of juveniles or their chances of recruiting into the population compared to older individuals (de Vries *et al*., 2023). On the other hand, if density dependence affects the survival of older individuals in a population, then slow life histories are favoured, contrary to Williams' hypothesis (Gaillard & Lemaître, 2017). Density dependence can affect survival or reproduction (either fertility or juvenile production) or both in an age‐dependent or age‐independent fashion. As a result, linking assumptions to outcomes is not straightforward, and population size might change over time in complex ways. The possibility that density dependence will change mortality and that, conversely, mortality can change density dependence, adds another layer of complexity that is not accounted for in Williams' hypothesis (Day & Abrams, 2020).

Monogyny is the ancestral, and most common condition found in ants, where a single reproductive queen starts a colony on her own (Hölldobler & Wilson, 1990; Ross & Carpenter, 1991). Young queens experience high mortality (Hölldobler & Wilson, 1990; Keeler, 2022; Gordon, 2024), but as the colony grows and becomes established this diminishes immensely. Slow life histories are predicted to evolve under this density‐dependence scenario with differential survival of younger and older queens. Furthermore, high competition in densely populated habitats can lead to density‐dependence effects on fertility, as younger queens in small colonies are easily outcompeted (Gordon, 2024). Colony founding *via* budding or fission is a derived character in ants and typical of polygynous species (Keller, 1991). Here, the mortality rates of young and older queens are less likely to differ dramatically and to favour slow life histories.

Modelling the effect of age‐independent extrinsic mortality in a superorganismal context showed that lifespan differences between castes can still evolve in the absence of extrinsic mortality. A model that implemented mutation accumulation in an age‐structured simulated population, and antagonistic pleotropic effects within and between castes, predicted that lifespan divergence evolves in the absence of extrinsic mortality (Kreider, Pen & Kramer, 2021). The model also predicted that between‐caste antagonistic effects have a stronger impact on worker lifespan than on queen lifespan (Kreider *et al*., 2021). This suggests that deleterious effects can be borne by workers rather than queens, enhancing the overall fitness of the colony (Kreider *et al*., 2021). A second model, also based on mutation accumulation, showed that the delayed production of sexuals and resource monopolization by reproductives of social insects are more important for the evolution of diverging lifespans than extrinsic mortality, with queens living longer (Kramer *et al*., 2022). A small but positive effect of extrinsic mortality on lifespan divergence was generally found, except in cases where reproduction was restricted to a single queen and workers were sterile (Kramer *et al*., 2022). This is opposite to the antagonistic pleiotropy model, where the authors found that lifespan differences were larger if workers were sterile (Kreider *et al*., 2021).

### (2) The role of reproductive strategy

In addition to the effect of extrinsic mortality, the shape and pace of ageing are determined by the reproductive strategy of a species. Cole (1954) introduced the terms “iteroparity” and “semelparity” to distinguish between species that reproduce repeatedly or only once, respectively. Currently there is a debate about whether parity strategies lie along a continuum between these two concepts (Hughes, 2017). Semelparity encompasses many species with very different life histories and litter sizes, for example salmon that migrate upstream to breed and then die (Gems *et al*., 2021), or the periodical cicada *Magicicada* that emerge as adults for only a short period of time after 13 or 17 years as nymphs (Sota, 2022).

**Fig. 1:**
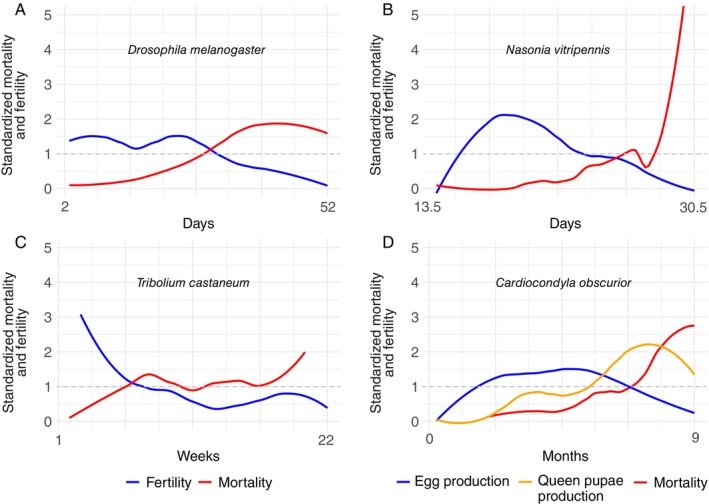
Life‐trajectories of four model insect species. Standardized age‐specific fertility and mortality shown up to the point when 95% of the population has died. The dashed grey line at *y* = 1 indicates when relative mortality or fertility are equal to mean mortality or fertility, respectively. (A) *Drosophila melanogaster* (Khazaeli & Curtsinger, 2010). (B) *Nasonia vitripennis* in the host *Lucilia sericata* (Chabora, 1970). (C) *Tribolium castaneum* data obtained using WebPlotAnalyzer (Rohatgi, 2023) using the average for females mated (to multiple and single males) once every 2‐, 4‐, 12‐ and 20‐weeks (Pai & Yan, 2020). (D) Production of eggs or of queen pupae and mortality in *Cardiocondyla obscurior* (Jaimes‐Nino *et al*., 2022a).

Similarly, iteroparity describes a broad range of shapes of ageing (Jones *et al*., 2014). For example, in Fig. 1 we compare the shape and pace of ageing of three widely used model insects (the fruit fly *Drosophila melanogaster*, a parasitoid wasp *Nasonia vitripennis* and the red flour beetle *Tribolium castaneum*), and our own model, the ant *Cardiocondyla obscurior*. *D. melanogaster* has relatively constant fertility over *ca*. 50% of their *ca*. 7.5‐weeks‐long life, at which point mortality increases above average (Khazaeli & Curtsinger, 2010; Fig. 1A). *N. vitripennis* exhibits a fast life‐history strategy: it is a short‐lived (2–4 weeks) parasitoid wasp that depends on *Protocalliphora* flies, parasites of birds (Chabora, 1970; Fig. 1B). Suitable host nests are patchily distributed in the environment (Mair & Ruther, 2019). Mortality increases sharply towards the end of their life but is unrelated to an early peak in fertility at 25% of their lifespan. The fertility of *Tribolium castaneum* declines to below average after 30% of their life (~8 weeks) (Pai, Bennett & Yan, 2005), and at the same point mortality increases above average (Fig. 1C). In *C. obscurior*, ant queens exhibit an above‐average standardized fertility (measured as egg production) for ~60% of their life from week 6 until week 28 (Fig. 1D). Mortality is below‐average until week 28, and queen pupae production reaches a peak at ~ week 30 (Jaimes‐Nino, Heinze & Oettler, 2022a; Fig. 1D).

**Fig. 2:**
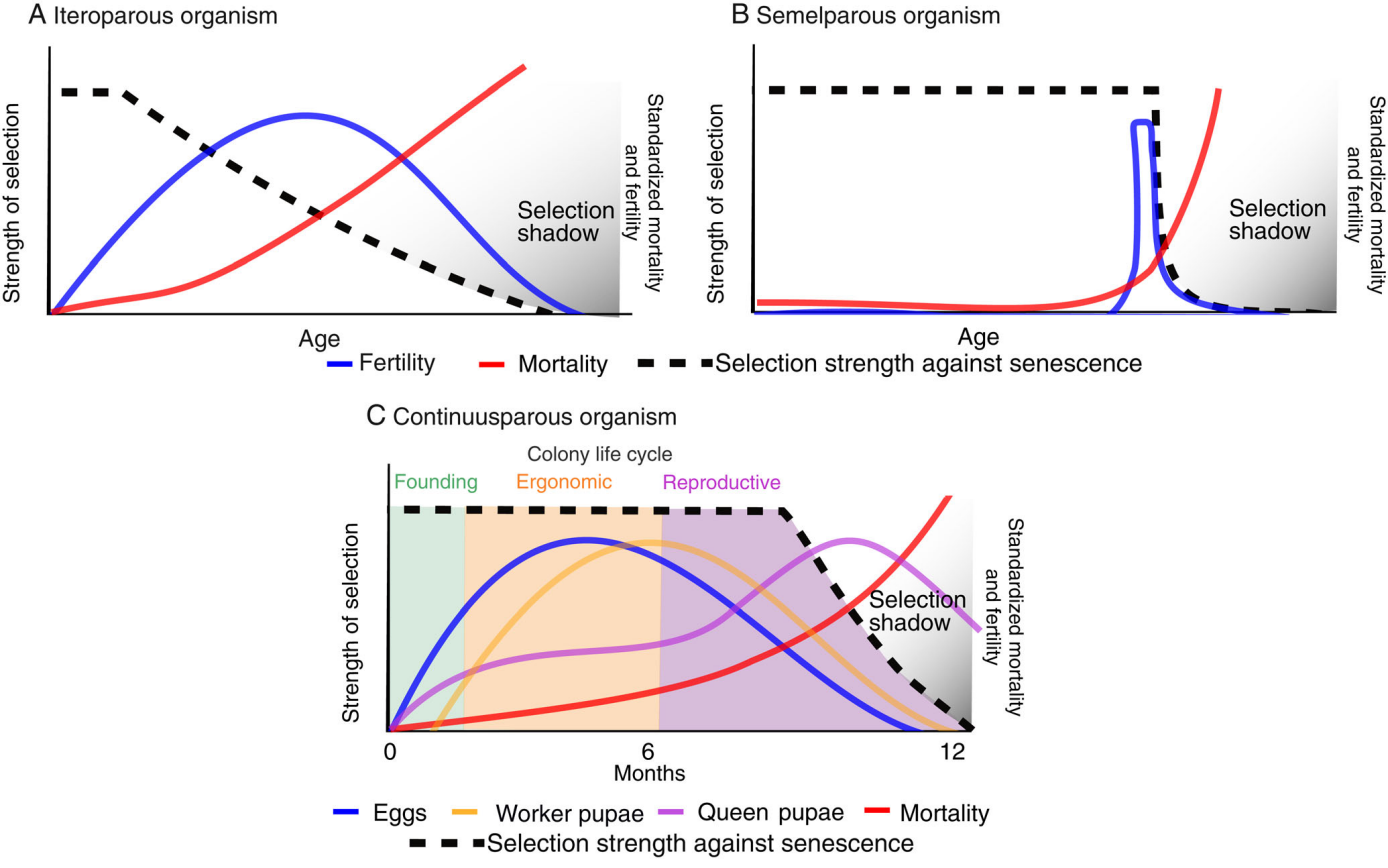
Comparison of the strength of selection (black dashed lines) against age‐specific mortality for different life history strategies: (A) iteroparity; (B) semelparity; and (C) continuusparity. Standardized age‐specific ant queen fertility and mortality are denoted by blue and red lines respectively. Continuusparity predicts that the selection strength is maximized in ant queens during the founding and ergonomic stage (worker production denoted by a yellow line), and fitness payoffs come only later in life (the reproductive phase with a maximum of queen pupae production, purple line) after the maximum investment into workers has been reached. Continuusparity is characterized by a delayed selection shadow, more comparable to the semelparous strategy.

While conceptually, a selection shadow can occur in iteroparous and semelparous organisms, as in both cases selection strength will decline following the onset of reproduction, these two strategies will differ in terms of the selection strength. In iteroparous species, selection strength declines more or less gradually after the first bout of reproduction (Fig. 2A), whereas in semelparous organisms it remains constant until a steep decline during a short period of rapid senescence with a sharp increase in mortality rate, that is post‐reproductive death (Fig. 2B; Finch, 1994). Ants are considered superorganisms, with selection acting at the colony level (Wheeler, 1986; Boomsma & Gawne, 2018). Most ant queens found a colony independently, followed by an ergonomic growth phase characterized by investment into worker production (Oster & Wilson, 1978) (Fig. 2C). In some taxa, the ergonomic growth phase can last between 3 and 8 years before the colony produces its first sexual offspring (Tsuji & Tsuji, 1996). Colony‐level reproduction (the onset of production of reproductives) begins either when the queen produces sexual offspring or when workers produce male offspring from unfertilized eggs in species with worker reproduction. In the case of species with sterile workers, as *C. obscurior*, the production of workers cannot translate into direct fitness returns, and colony‐level reproduction equates to queen (or queens if there is more than one) individual‐level reproduction.

While colony‐level production can start in the early phase of colony growth, queens experience greater fitness returns with the production of sexuals later in the life of the colony, with consequences for the selection shadow (Fig. 2C). The shape of ageing of *C. obscurior* queens, one of the best‐studied ant species regarding lifespan variation (Jaimes‐Nino *et al*., 2022a, 2022), combines characteristics of both a semelparous and an iteroparous strategy (Fig. 2). The production of sexual offspring starts early in life, approximately 4 weeks after mating, and gradually increases with age (thus these queens are iteroparous). However, mortality increases only late in life close to the peak of sexual production at week 30 (Jaimes‐Nino *et al*., 2022a), and both observations and gene expression data indicate that queens exhibit a long healthspan (the period of life without clear signs of senescence) (Wyschetzki *et al*., 2015; Harrison *et al*., 2021; Jaimes‐Nino *et al*., 2022a; Jaimes‐Nino & Oettler, 2025), in a pattern more like that of semelparity (Fig. 2).

This has led us to propose the term “continuusparity” (from Latin *continuus* meaning “incessant/successive” and *parere* meaning “giving birth”). Continuusparity describes the repeated reproduction of an organism while maintaining the strength of selection against senescence due to late‐life fitness gains. Continuusparity differs from iteroparity, where the strength of selection against senescence declines with age, after the first reproductive event. Such a reproductive strategy is applicable to eusocial species with reproductive division of labour and describes the pattern where fitness returns increase with age even after the peak of egg production and after the onset of production of sexuals. Selection strength is maintained, resulting in a delayed selection shadow and the onset of actuarial senescence (Jaimes‐Nino *et al*., 2022a). Continuusparity is not to be confused with negligible senescence, as queens do exhibit clear signs of reproductive and actuarial senescence but only for a very short period (~2 weeks or 4% of maximum lifespan) (Harrison *et al*., 2021; Jaimes‐Nino *et al*., 2022a). Continuusparity might occur in other eusocial organisms. For example, the naked mole rat *Heterocephalus glaber* is known for delayed or reduced age‐associated physiological decline (Lewis & Buffenstein, 2016). Once a female becomes a breeder, she can reproduce continuously (~ every 3 months) until she dies (Urison & Buffenstein, 1995). Remarkably, even at ages 25‐fold their age to reproductive maturity, age‐specific mortality does not increase (Ruby, Smith & Buffenstein, 2018). Breeding females are fertile well into their third decade of life and seem to exhibit an increase in reproductive output with age (Buffenstein, 2008). The continuusparity framework predicts that the strength of selection against senescence is maintained in old, well‐established breeders if they produce larger litter sizes than those of younger breeders. Data on age‐specific fertility are needed to confirm this hypothesis.

In principle, senescence can be selected against, even after the reproductive peak. One example is the “grandmother hypothesis” (Hill & Hurtado, 1991), which explains the extended post‐reproductive lifespan of human females as resulting from fitness payoffs from caring for relatives. This hypothesis was supported by comparative studies in toothed whales, where menopause evolved independently several times, illustrating the evolution of lifespan extension after the reproductive phase (Ellis *et al*., 2024). While there may be some analogy to an extended lifespan due to late‐life fitness returns in ant queens, the fitness returns of delaying the onset of actuarial senescence in ant queens result from direct rather than indirect fitness because reproduction does not cease. It would be informative to investigate whether, in ants and other eusocial species, the evolutionary mechanisms counteracting actuarial senescence are similar to those acting against premature senescence in semelparous species, and in iteroparous species with menopause after reproduction has ceased.

It is unclear exactly how the social environment helps ant queens to minimize the reproduction–longevity (= somatic maintenance) trade‐off. For most species, one of the most fundamental trade‐offs is between early fecundity and late fecundity. However, in social insects an initial investment into workers pays off during later phases of production of reproductives, thus early and late fecundity are intricately positively linked. Further, the need for investment into costly larval traits associated with competitiveness over resources is much reduced in a eusocial environment, where resources are optimized at the colony level and competition between larvae is predicted to be rare (Schultner, Oettler & Helanterä, 2017). This is especially true in a species like *C. obscurior* in which conflict between queens and workers over reproduction seems to be completely absent (Schultner *et al*., 2023) but might vary among social species where direct fitness gains are relevant and workers can reproduce. Finally, the indirect costs of reproduction (food provisioning, nest construction, and defence) are borne by the workers alone – a unique aspect of superorganismality.

### (3) The shape of ageing in other superorganisms

**Fig. 3:**
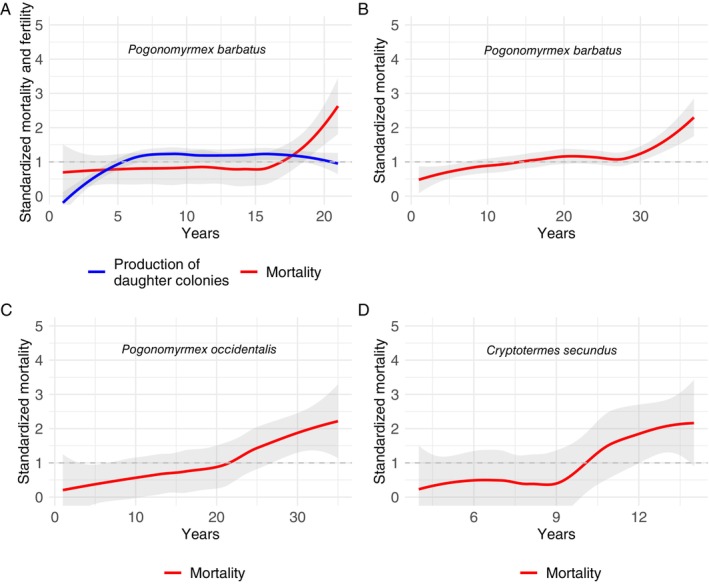
Standardized mortality of long‐lived *Pogonomyrmex* ant and *Cryptotermes* termite queens. Relative mortality is shown up to the point when 95% of the population has died. The dashed grey line at *y* = 1 indicates when relative mortality (or fertility in terms of production of daughter colonies in A) is equivalent to mean mortality (or fertility in A). Note that mortality at the founding stage was not recorded. (A) Data from a long‐term (28 years, 1985–2013) study of *Pogonomyrmex barbatus* (Ingram *et al*., 2013), the standardized fertility was based on assessment of the parental status of 265 colonies (see text for details). (B) Data from A with additional data from the same population (*N* = 1057 colonies) in western New Mexico conducted from 1988 to 2019 (Sundaram *et al*., 2022). (C) Data for *P. occidentalis* (*N* = 112) in western Nebraska (Keeler, 2022). (D) Data from a laboratory study of *Cryptotermes secundus* termite queens (*N* = 41) (Monroy Kuhn *et al*., 2021).

In addition to *C. obscurior*, sufficient demographic data are available for two species of North American *Pogonomyrmex* seed‐harvester ants from which we could calculate ageing trajectories. One long‐term study monitored 300 individual colonies of a population of *Pogonomyrmex barbatus* from 1985 to 2013 (Ingram *et al*., 2013). Reproductive success of queens was estimated from the number of daughters that successfully founded new colonies in a 20 × 400 m area (Ingram *et al*., 2013). Mother–daughter colony pairs were identified using microsatellite markers to assess relatedness and to calculate parentage exclusion probabilities for 265 colonies (Ingram *et al*., 2013). The production of daughter colonies did not decrease with time (Fig. 3A), suggesting that these ant queens did not exhibit reproductive senescence. It is important to corroborate if their relative realized reproductive success reflects their potential relative reproductive success, given the mean dispersal distance for colony founding of 150 m (Ingram *et al*., 2013), meaning that additional colonies may have been initiated outside the study site. Age‐dependent mortality of a larger data set (*N* = 1057 colonies) including the same *P. barbatus* population (Sundaram, Steiner & Gordon, 2022) followed a similarly shaped standardized mortality curve, with low and constant mortality for up to 75% of the queen's lifespan (Fig. 3A, B), increasing steeply only late in life. Another study marked and monitored 112 colonies of a population of *Pogonomyrmex occidentalis* in Nebraska (Keeler, 2022). In this case, the standardized mortality seems to be lowest during the first 25% of the lifespan but increases more steeply relatively earlier than in *P. barbatus* (Fig. 3C).

Demonstrating potential similarities due to a transition to superorganismality, queen ageing in the long‐lived termite *Cryptotermes secundus* under controlled laboratory conditions again is non‐gradual, with a delay in the onset of actuarial senescence until 70% of queen lifespan, at around 10 years (Fig. 3D; Monroy Kuhn, Meusemann & Korb, 2021).

## III. WHY AND HOW DO SPECIES VARY IN THE PACE OF AGEING?

### (1) The pace of ageing in ants

Ants provide an interesting opportunity to study lifespan variation. A comparison of maximum lifespans of ant queens from 51 species (representing 23 of about 300 extant ant genera; Bolton, 1995) with 81 solitary insect species reported an astonishing 100‐fold difference in lifespan (mean ± SD, 10 ± 6.6 years *versus* 0.1 ± 0.2 years, respectively) (Keller & Genoud, 1997). However, much of the information used to derive these values is anecdotal or difficult to trace. For example, the existence of a 28‐year‐old queen that has been repeatedly cited in the scientific (Keller & Genoud, 1997; Keller, 1998; Fjerdingstad & Keller, 2004; Jemielity *et al*., 2005; Gräff *et al*., 2007; Parker, 2010; Kramer, Schaible & Scheuerlein, 2016; Pamminger *et al*., 2016; Lucas & Keller, 2018; Schläppi *et al*., 2020) and general literature (Law, 2021) is difficult to substantiate. The source, an obituary of the amateur myrmecologist Hermann Appel, reads: “A *Lasius niger* ant queen can live for almost 30 years. We owe this sensational statement precisely to H. Appel. He left the following note about it: ‘The ant queen (*Lasius niger*) was caught in August 1931 after the nuptial flight; she lived with me in captivity until April 1950 (i.e. 28 3/4 years!)’” (Kutter & Stumper, 1969, p. 279). But 1950–1931 does not equal 29, and it is impossible to determine whether this was an error in the reported dates, a miscalculation, or a typo.

**Fig. 4:**
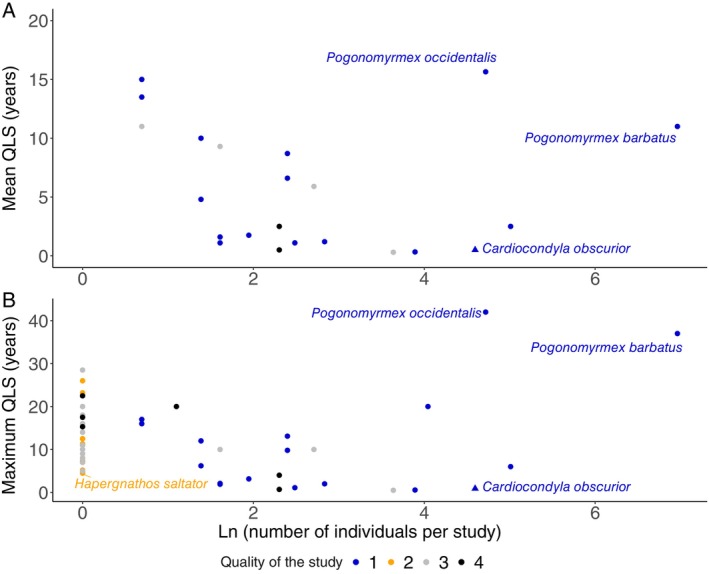
Reported mean (A) and maximum (B) ant queen lifespan (QLS) plotted against number of individuals (on natural logarithmic scale) for which the estimate was obtained. Data are taken from Table S1. A quality score (1–4) was assigned to each study, where 4 (black circles) indicates study not accessible; 3 (grey circles) is used for data cited as “personal communication”, where the original text did not mention the reported lifespan, or lifespan was estimated without reporting the number of observed individuals; 2 (orange circles) is *N* = 1 observed individual; and 1 (blue circles) is *N* > 1.

A search in *Web of Science* retrieved 802 studies (all databases, accessed 05.07.2024) using the terms [(longevity OR lifespan) AND (ant OR Formicidae)] (see online Supporting Information, File S1). We filtered the results to include only those that reported queen lifespans and added additional studies known to us (studies cited in Kramer & Schaible, 2013), plus data from *Pogonomyrmex barbatus* (Sundaram *et al*., 2022) (Table S1). From the 78 filtered studies, 52 (67%) relied on data from a single or an undocumented number of individuals (Table S1, Fig. 4B). Taking only one species per genus to avoid pseudo‐replication (the ant species with the longest reported lifespan), removing inaccessible studies or data from personal communications, and including only studies with a sample size >1, resulted in a short list of nine species. From these data we calculated a median maximum queen lifespan of 7.9 years (range 0.9–20 years). Given that data on longer‐lived species are harder to obtain, this is likely an underestimate. However, even with a bias towards studying short‐lived species, we still identify a range of three orders of magnitude in queen lifespan, supporting the suggestion that the massive diversity in ageing found in ants has potential to illuminate our understanding of both ultimate and proximate aspects of ageing.

While several species of *Pogonomyrmex* stand out with a maximum lifespan of 30 years or more (Tables 1 and S1), we still are far from being able to generalize that ant queens on average live longer than solitary insects. This will only be possible when extensive data, more representative of the broad diversity of the 14,000 described Formicidae species and of the million solitary insect species, become available. We note that the mean lifespan reported by Keller & Genoud (1997) for solitary insects did not include some potentially long‐lived representatives of Odonata, Megaloptera, Belostomatidae, cicadas of the genus *Magicicada*, giant wetas from the genus *Deinacrida*, Phasmatodea, hissing cockroaches such as *Gromphadorhina portentosa*, and Buprestidae beetles. Additional data are needed, particularly based on cohorts with sufficient replicates, to confirm that ants do have “extraordinary lifespans” (Keller & Genoud, 1997).

**Table 1:** Selected reported mean and maximum ant queen lifespan (QLS). The complete list of all species for which we could locate data can be found in Table S1.

Species	Mean QLS (years)	Max QLS (years)	Number of individuals	Reference
*Cardiocondyla obscurior*	0.5	0.9	99	Jaimes‐Nino *et al*. (2022a)
*Harpegnathos saltator*				
gamergates*	3.02	5.4	55	Peeters *et al*. (2000); Ghaninia *et al*. (2017)
workers^†^	0.6	1	61	Ghaninia *et al*. (2017)
queens		5.2	1	Peeters *et al*. (2000)
*Pogonomyrmex barbatus*	11	37	1057	Sundaram *et al*. (2022)
*Pogonomyrmex occidentalis*	15.65	42	112	Keeler (1993, 2022)

* Egg‐laying worker (gamergate) lifespans.

^†^ Worker lifespans.

Similarly imprecise is the comparison of queen lifespans between 37 monogynous and six polygynous species (mean ± SD 12.3 ± 5.5 *versus* 1.6 ± 1.8 years, respectively) (Keller & Genoud, 1997). Given the low replicate numbers involved, these values remain questionable. Future studies should take into account life‐history traits, such as phylogeny, body size, colony size, habitat, colony founding strategy, ecozone, and the timing, seasonality, and value of investment into workers and sexuals, which all may affect the evolution of ageing. Ants provide an exceptional range of subjects for experimental investigation. Several genera show variations in social structure suitable for conducting comparative analyses. Species pairs with monogynous and polygynous populations that could be tracked and compared, occur in *Formica, Iridomyrmex, Leptothorax, Myrmica, Monomorium, Neivamyrmex, Plagiolepis, Pogonomyrmex, Solenopsis, Tapinoma* (Mackay *et al*., 1990; Overson, Fewell & Gadau, 2016), and probably several others as well. Such data could provide an answer to the question of whether, and why, polygynous queens have shorter lifespans.

### (2) The long lifespans of seed harvester ants

Queen *P. occidentalis* and *P. barbatus* seed harvester ants currently set the longevity record, at 42 and 37 years, respectively (Keeler, 1993, 2022; Sundaram *et al*., 2022). Both species occur at high densities in arid environments (Hölldobler, 1976; Sundaram *et al*., 2022; Keeler, 2022). Summer rains trigger mass emergence of winged sexuals which meet in large mating aggregations. Mated queens fly off, and shed their wings upon landing, whereupon they search for a suitable nest site, dig into the soil, and produce a first small batch of workers, either claustrally, that is from their fat body reserves, or with the aid of energy from foraging (semi‐claustral colony founding). It takes colonies a few years to reach a size/age threshold at which they produce sexuals, but once a colony reproduces it continues to do so, although not every year if conditions are suboptimal (Cole & Wiernasz, 2000). While independently founding queens of other species might experience similar scenarios, in the case of *Pogonomyrmex* spp., differences in age‐dependent extrinsic mortalities may be extreme. Colonies vary in their foraging behaviour with age, becoming more effective at securing their foraging area against neighbouring colonies (Gordon, 2024). It is likely that a combination of high density‐dependent extrinsic mortality during mating flights and colony foundation, coupled with the low mortality of long‐established mature colonies in a stable habitat (Gordon, 2024), explains the selection for extended colony and queen lifespans.

## IV. WHY DOES AGEING VARY WITHIN TAXA?

A third question of interest is why some individuals in a population live longer than others. Ants were proposed as promising models to study this question, because queens have been assumed to have much longer lifespans than workers (Hölldobler & Wilson, 1990; Giraldo & Traniello, 2014; Korb & Heinze, 2021; Kramer *et al*., 2022). The most comprehensive study in support of this assumption collected ant queen mean/maximum lifespan data from 36/47 species and worker data from 27/33 species (Kramer & Schaible, 2013). However, a closer look at their data reveals that in some cases the workers were not part of age‐controlled cohorts, and sometimes the data are ambiguous. For example, the worker lifespan estimate for *Atta colombica* was deduced from the disappearance of workers sprayed with fluorescent ink in a field study (Porter & Bowers, 1982), confounding extrinsic and intrinsic mortality rates. Furthermore, the age at which workers began leaving the nest to forage in this study was unknown, but studies in other species have demonstrated that ant workers can initiate foraging at any age (Oettler & Johnson, 2009; Oettler, Nachtigal & Schrader, 2015), thus such observations cannot be considered robust evidence of worker lifespans. Filtering the data (to one species per genus, retaining the species with the largest age, *N* > 1 for both queens and workers) results in a short list of only three species, of which one, *Cardiocondyla obscurior*, does not exhibit diverging lifespans. The remaining two, *Diacamma rugosum*, and *Myrmecia vindex*, show a fourfold greater queen lifespan than that of workers (Table S1). In the case of *Diacamma rugosum*, a queenless species, dominant individuals mate and reproduce. The reproductives were compared to non‐reproductives which were marked with enamel paint; the authors attributed the early death of workers due to handling (Tsuji, Nakata & Heinze, 1996).

Other than age‐independent extrinsic mortality (Oster & Wilson, 1978), which has been shown to lack strong theoretical support (see Section II.1; Kreider *et al*., 2021; Kramer *et al*., 2022), it was hypothesized that variation in lifespan could be due to differences in fertility among individuals (Lin & Michener, 1972; Hartmann & Heinze, 2003; Heinze, Frohschammer & Bernadou, 2013; Dixon, Kuster & Rueppell, 2014; Fuessl, Heinze & Schrempf, 2015; Kramer *et al*., 2015; Heinze & Giehr, 2021; Negroni *et al*., 2021). *Harpegnathos saltator*, for instance, is characterized by a prolonged colony lifespan, past the death of the founding queen. Workers are slightly smaller than the winged queen morph and have a similar reproductive anatomy, with 4 + 4 ovarioles and a spermatheca, but they are only half as fertile (Peeters, Liebig & Hölldobler, 2000). It has repeatedly been shown that being the reproductive in a colony is positively correlated with lifespan extension in ponerine ants with totipotent workers (Tsuji & Tsuji, 1996; Hartmann & Heinze, 2003), and *H. saltator* is no different. Workers can mate and begin reproducing after the queen dies, once ritualized tournaments have established a dominance hierarchy. The drastic changes in physiology and lifespan when a worker transitions to become an egg‐laying worker, called a “gamergate”, have been well documented (Sheng *et al*., 2020; Yan *et al*., 2022; Glastad *et al*., 2023). The colony lifespan of *H. saltator* in the field is thought to be limited to one succession, and only queens and not workers/gamergates seem to found new colonies (Peeters *et al*., 2000). Workers live less than 1 year in the laboratory, but gamergates can live for 3–5 years [mean for workers is 219 days (*N* = 61) and for gamergates is 1103 days (*N* = 55); Ghaninia *et al*., 2017]. Gamergates apparently do not live for as long as queens (Table 1, 5.2 years; Peeters *et al*., 2000), although this queen age estimate was based on a single individual. Whether lifespan changes in totipotent workers are caused by fertility or dominance cannot be easily disentangled.

For *C. obscurior*, which seems to contradict the claim of diverging queen and worker lifespans, workers and queens have a similar standardized mortality (i.e. age‐specific mortality standardized by mean mortality), median and maximum lifespan (Table S1; Jaimes‐Nino *et al*., 2022b), although the two castes differ in fertility. Additionally, in this species queen lifespan and fertility may not be causally linked (Schrempf, Heinze & Cremer, 2005; Will *et al*., 2012; Oettler & Schrempf, 2016). Increasing queen fertility *via* egg removal (Schrempf *et al*., 2017), and manipulation of investment into sexual production *via* changes to colony size (Jaimes‐Nino *et al*., 2022a) did not affect the lifespan of queens of *C. obscurior*. These observations suggest that fertility does not affect the rate of ageing in this species.

## V. DISCUSSION AND FUTURE DIRECTIONS

The advantages offered by studying social insects as model organisms for ageing research have been outlined elsewhere (Heinze & Schrempf, 2008; Korb, 2024). Here we review the available data and try to infer the causes and consequences of superorganismal ageing. We conclude that ants combine a benefit of eusociality, that is late‐life fitness returns, with properties of both itero‐ and semelparity, that is repeated reproduction but a very short selection shadow (Fig. 2). Several aspects remain to be explored.

### (1) Reproductive death

The lack of signs of senescence in elderly *C. obscurior* queens (Harrison *et al*., 2021; Jaimes‐Nino & Oettler, 2025), the sharp increase in mortality in late age, and gene expression patterns indicative of massive pathologies that take place a few days before queens die (Jaimes‐Nino *et al*., 2022a) suggest that death may be a consequence of reproductive effort. Moreover, time course and co‐expression network analyses of gene expression patterns over time in the termite *Cryptotermes secundus* showed that queens exhibit a non‐gradual ageing pattern with sudden death at 11–13 years, characterized by a strong molecular signal indicating the loss of proteostasis (Monroy Kuhn, Meusemann & Korb, 2021).

The phenomenon of reproductive death is common to semelparous species, which undergo a massive translocation of resources at the time of reproduction (Young & Augspurger, 1991). For example, in *Caenorhabditis elegans* hermaphrodites, reproduction ceases after 3 days of adulthood and is followed by reproductive death, during which yolk is vented to be consumed by larval progeny (Kern & Gems, 2022; Kern *et al*., 2023). The pathologies developed are caused predominantly by hyperfunction of developmental programs rather than by molecular damage (Ezcurra *et al*., 2018; Sornda *et al*., 2019). Due to this type of adaptive death with clear fitness benefits (Lohr, Galimov & Gems, 2019), among other traits, *C. elegans* hermaphrodites were proposed as semelparous (Gems *et al*., 2021). On the other hand, quasi‐programmed ageing, that is a continuation of a developmental program that is not turned off and is without fitness benefits, could be seen as a mechanism for senescent pathologies in iteroparous organisms (Blagosklonny, 2006; de Magalhães, 2012). A distinction can be made between continuation of a futile program and resource allocation that translates into fitness gains. In the case of *C. elegans*, reproductive death occurs as a combination of both quasi‐programming and adaptive death. It remains to be shown whether reproductive death is present in ants, what causes it, and, if it is triggered by terminal investment, how it can be adaptive despite ants reproducing continuously.

### (2) Colony‐level senescence

Above, we have discussed the shape of ageing in female reproductives of ants, without much focus on the shape of ageing at the colony level. In species with single‐queen colonies that do not recruit new queens after the queen's death, colony‐level senescence should mirror individual‐level senescence, as is the case in *P. barbatus* (Sundaram *et al*., 2022) and *P. occidentalis* (Keeler, 2022). In species where queens are continuously replaced, as in *C. obscurior* or the argentine ant *Linepithema humile*, colonies are theoretically immortal, similar to a non‐senescent *Hydra* colony. The same applies to monogynous colonies with colony inheritance, also known as serial polygyny, observed in *Nothomyrmecia macrops* (Sanetra & Crozier, 2002), and *Diacamma cyaneiventre* (Andre, Peeters & Doums, 2001). However, whether such colonies are indeed non‐senescent remains to be verified.

### (3) Call for more data

More lifetime mortality and fertility data are needed to corroborate whether delayed actuarial senescence and a lack of reproductive senescence are common in ants, even if such data are difficult to obtain, especially for long‐lived species. For example, a 10‐year study on *Formica exsecta* deduced a lifespan of 20 years (Pamilo, 1991) but no data were collected on age‐dependent mortality for the final period of life that will be needed to estimate age‐specific standardized mortality and lifespan. For verification of extended lifespans, studies on age‐controlled cohorts, with appropriate replication, and on species representative of other ant subfamilies are needed. However, ants are not only diverse but also notoriously difficult to rear in captivity, making data acquisition challenging. Lastly, research is needed to understand the conditions under which queen and worker lifespans diverge and why, as lifespan does not seem to be directly related to fertility. Ideally, comparable survival and fertility data should originate from the same study, as ageing trajectories from different assays or environmental conditions can affect not only the temporal scaling but also the shape of ageing trajectories.

## VI. CONCLUSIONS

(1) Data confirming that the lifespans of ant queens are “extraordinary” are available for only a few species. Data showing that monogynous queens live longer than polygynous queens are scarce.
(2) The trajectory of ageing in ants is a consequence of both reproductive strategy and age‐dependent extrinsic mortality. The effect of age‐independent extrinsic mortality in ants requires further study to account for density‐dependence scenarios.
(3) Continuusparity is a term introduced to describe the repeated reproduction of an organism while maintaining the strength of selection against senescence due to late‐life fitness gains. Continuusparity differs from iteroparity, where the strength of selection against senescence declines after the first reproductive event. The similar shape of ageing in ants and a termite species suggests similarities across independent evolutionary transitions to superorganismality.

## Supporting information


**File S1.** Results from *Web of Science* search on all databases retrieved on 05.07.2024 using the search string [(longevity OR lifespan) AND (ant OR Formicidae)] (.ris file).

**Table S1.** Reported queen and worker lifespans (Formicidae) from publications identified by our literature search or from additional articles known to us.
